# MicroRNA degeneracy and pluripotentiality within a Lavallière-tie architecture confers robustness to gene expression networks

**DOI:** 10.1007/s00018-016-2186-1

**Published:** 2016-04-01

**Authors:** Ricky Bhajun, Laurent Guyon, Xavier Gidrol

**Affiliations:** 1 CEA, BIG, BGE, 17, rue des Martyrs, 38000 Grenoble, France; 2 University Grenoble Alpes, BGE, 38000 Grenoble, France; 3 INSERM, U1038, 38000 Grenoble, France

## Abstract

Modularity, feedback control, functional redundancy and bowtie architecture have been proposed as key factors that confer robustness to complex biological systems. MicroRNAs (miRNAs) are highly conserved but functionally dispensable. These antinomic properties suggest that miRNAs fine-tune gene expression rather than act as genetic switches. We synthesize published and unpublished data and hypothesize that miRNA pluripotentiality acts to buffer gene expression, while miRNA degeneracy tunes the expression of targets, thus providing robustness to gene expression networks. Furthermore, we propose a Lavallière-tie architecture by integrating signal transduction, miRNAs and protein expression data to model complex gene expression networks.

## Introduction

MicroRNAs (miRNAs) are small non-coding RNAs that repress gene expression post-transcriptionally. These RNAs represent 1–2 % of all genes in metazoans and are highly conserved between species [10]. More than 2588 known mature human miRNAs are listed in the 21st release of the miRBase [24]. Each miRNA is predicted to regulate between one dozen and thousands of genes, and most human protein coding genes (approximately 60 %) are susceptible to regulation by several miRNAs [43]. Numerous studies have demonstrated the crucial role of miRNAs in cell and organ physiology as well as in human diseases. miRNAs control cell proliferation, cell differentiation, organ development and tissue homeostasis. Animals carrying a loss-of-function mutation in the miRNA machinery are not viable, which indicates that miRNA activity is indispensable to life [13, 18, 39].

In contrast to their demonstrated importance as key regulators of gene expression, individual miRNA knockout animals exhibit very modest or no apparent phenotype [37]. In *Caenorhabditis elegans,* less than 10 % of miRNAs are individually required for normal development or viability [26], which seems to also be true in mice [29, 37]. So far, only two miRNA genes (miR-17~92 and miR-96) seem to cause developmental defects in humans when mutated [7, 28]. The emerging view is that rather than acting as key genetic switches, miRNAs are similar to rheostats, synergistically and finely tuning the expression of hundreds of protein-coding genes to reinforce the cell fate triggered by other mechanisms [3, 8, 18].

Robustness is defined as the capacity of biological systems to maintain specific functions when exposed to internal or external perturbations [23]. These properties suggest the important role of miRNAs in providing robustness to biological systems. As recently discussed in a review by Ebert and Sharp [8], this result is further demonstrated by the following observations: “(1) genes with tissue-specific expression have longer 3′ UTRs with more miRNA-binding sites [33]; (2) miRNA expression increases and diversifies over the course of embryonic development [34], as 3′ UTRs are lengthened via alternative polyadenylation site choice [19]; and (3) the diversity of the miRNA repertoire in animal genomes has increased with increasing organismal complexity [17, 25]”. The underlying properties that provide robustness to complex biological systems have been studied in different models and are mainly modularity, bowtie architectures and functional redundancy [35, 40, 41].

Here, to understand the complexity of miRNA regulation, we further evaluated whether the human miRNA network that we recently characterized [1] exhibits some of these properties to increase robustness in gene expression.

## Does the human miRNA network exhibit modularity and bowtie structure?

**Fig. 1:**
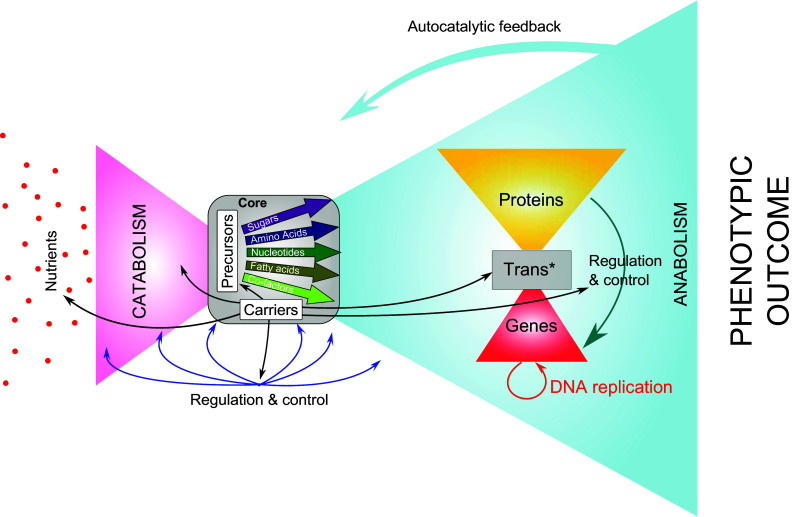
Bowtie architecture of metabolic networks. This is a modified Figure from Csete and Doyle [6]. Bowtie architecture is a combination of two modules coupled via a central element or core, which is defined as a reduced set of building blocks. All modern technologies, from manufacturing to the Internet, are organized with bow ties architecture. On the *left hand* side of the structure (pink wing), nutrients are catabolized into a few precursors (sugars, amino acids, nucleotides, fatty acids, and co-factors) that will be used as energy and building blocks for the cellular anabolism on the *right hand* side of the structure side (*blue* wing). The whole process is tightly regulated and controlled through different systems (*black* and *blue arrows*). In the “anabolism” wing (*blue* wing) the gene expression network structure is represented. It also exhibits bowtie architecture, organized around a core the Trans* (transcription/translation) machinery composed of few polymerases and universal components enabling gene transcription and translation

A modified version of the bowtie organization, as defined by Csete and Doyle to model microbial metabolism [6], is presented in Fig. 1. In that structure, a myriad of nutrients are “fanned in” through a catabolic funnel on the left-handed side of the structure, to produce precursors and building blocks (e.g., amino acids, nucleotides, fatty acids, and sugars). The building blocks serve as a common currency between both sides of the bowtie. From the core of the bowtie, these blocks fan out into macromolecule synthesis by polymerase enzymes on the right-hand side of the structure. The macromolecular synthesis network also exhibits a bowtie structure (Fig. 1, right part). In this organization, genes fan in, whereas proteins fan out. At the center of this structure, a few evolutionarily conserved enzymes (i.e. DNA polymerases and RNA polymerases) and other components of the transcription/translation machinery (Trans*) compose the core, enabling the recycling of the building blocks, such as ribonucleotides and amino acids. Interestingly the bowtie organization makes robustness and evolvability compatible, which is a major characteristic of biological systems.

**Fig. 2:**
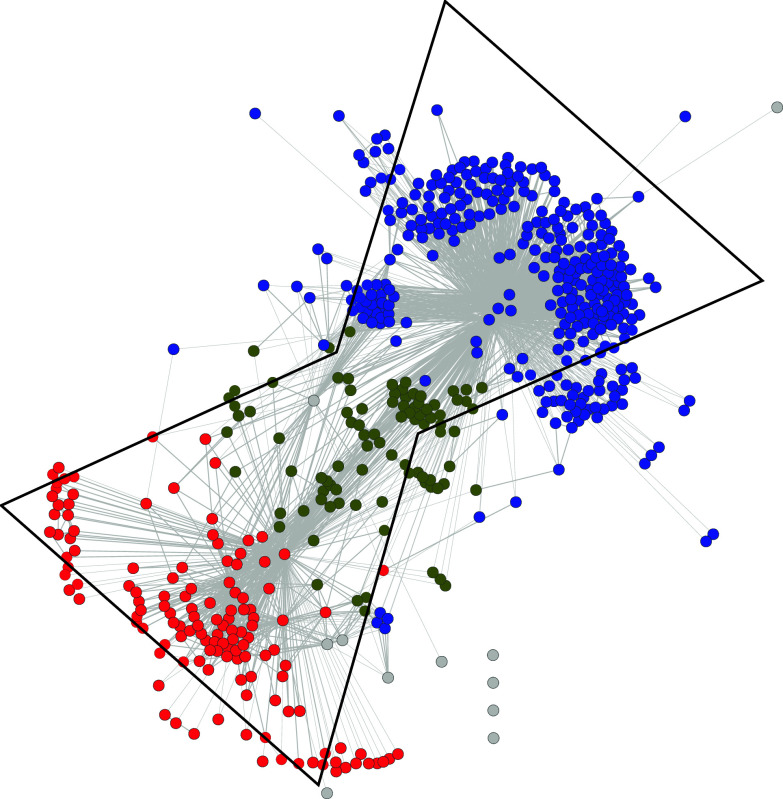
The miRNA network. This *graph* represents the underlying organization, of the miRNA network we have recently characterized [1]. The community of miRNAs in red or module 2 (lower part of the network), targets primarily protein-coding genes involved in signal transduction, particularly small GTPase signaling, would fan in information from the cell microenvironment. The *upper* part of the network, the *blue* community or module 1, regroups miRNAs that primarily target transcriptional regulators, fanning out gene expression toward diverse phenotypic outcomes in response to input signals. Central to the network, several miRNAs (*olive colored* nodes) are connected to either community but do not target any particular function in the cell. The modularity of this network and its underlying organization resemble bowtie. However, the nature of the core (*olive colored* nodes) composed of miRNA and not of a reduced set of building blocks, does not fit the definition of bowtie architecture [6]

We recently inferred a miRNA network based on target similarities among miRNAs [1]. Using the “DIANA-microT v3 (July 2009)” prediction database [22], we built a network where each node corresponds to a miRNA and each edge corresponds to the proportion of shared targets between two miRNAs. We used the meet/min index to infer the strength of the edge between two miRNAs [14, 16]. This index has a value between 0 and 1, where 1 implies that one miRNAs share all targets of the other miRNA and 0 implies no common target. At a meet/min threshold of 0.5, imposing the condition that two miRNAs are connected in the graph only if they share 50 % of their targets, we observed that the graph is comprised of two modules that are organized around a smaller one (Fig. 2) [1]. Our observation suggests that the miRNA network exhibited bowtie architecture, which was also observed using three other algorithms, namely TargetScan [15], miRanda [20] and PITA [21] (data not shown). The community of miRNAs in red or module 2 (Fig. 2, lower parts of the network), which targets primarily protein-coding genes involved in signal transduction, particularly small GTPase signaling, would fan in information from the cell microenvironment. The upper part of the network, the blue community or module 1, regroups miRNAs that primarily target transcriptional regulators, fanning out gene expression toward diverse phenotypic outcomes in response to input signals. Central to the network, several miRNAs (olive colored nodes) are connected to either community but do not target any particular function in the cell (Fig. 2).

However, as defined by Csete and Doyle [6], a bowtie can be interpreted as a combination of two modules coupled via a central element or core, which is defined as a reduced set of building blocks. Modularity in the network and shared protocols, that is “rules and interfaces by which module interacts [6]”, facilitates the recycling of building blocks within the network. In fact, the core (olive colored nodes) of the miRNA network in Fig. 2 is not composed of building blocks acting as common currency between the two modules, but rather of other miRNAs. Thus, although the human miRNA network that we inferred exhibits modularity, we do not believe that it fits the definition of bowtie architecture. We rather believe that this network is part of a larger gene expression network within the cell.

## Does the miRNA network exhibit functional redundancy?

MicroRNAs possess two characteristic features: degeneracy and pluripotentiality. Degeneracy refers to “the ability of structurally different elements of a system to perform the same function” [9, 11, 41]. Although often confused with redundancy, the concept of degeneracy is quite different from that of redundancy. While redundancy refers to the “one structure-one function” concept, degeneracy refers to “many structures-one function”. As already suggested by Edelman and Gally with the genetic code, miRNAs are a perfect example of degeneracy, as one given gene ensuring one function in the cell may be regulated by many different miRNAs with different nucleotide sequences. Redundancy of function within a system confers robustness to that system; thus, the ability to cope with unpredictable variation with minimal impact on system functionality. Degeneracy can provide even more robustness, such that if one element fails, a degenerate element can compensate that loss to conserve functionality (by analogy, when GPS fails because of a lack of power, an old paper map is always welcome). Furthermore, degeneracy has another advantage over redundancy, particularly for biological systems, such that degeneracy enables evolvability [9, 41]. Indeed, degenerate systems have a flexibility that makes them capable of developing new functionalities that may confer an evolutionary advantage to the biological system. The integration of degenerate miRNAs in gene expression network allows adjustment of expression to control cell fate in response to a wide range of conditions and environmental perturbations.

In contrast to degeneracy, pluripotentiality refers to a one function-many structures paradigm (e.g., a given kinase can phosphorylate dozens of proteins to ensure diverse cellular functions). Because a particular miRNA may recognize hundreds of different targets with different structures, miRNAs also exhibit pluripotentiality. For instance and based on the latest release of Tarbase (v7, date of access: 08/24/15) [31], let-7e-5p targets 2100 different validated genes in the human genome.

We further analyzed the degeneracy and pluripotentiality of each module in the human miRNA network that we have characterized (Fig. 2). The properties of each module in the network are reported in Table 1. The important values in this table are ratios A, B, and C; the larger the ratios are, the more targeted genes, and thus the greater pluripotentiality of the miRNA under scrutiny. In contrast, the smaller the ratios are, the fewer targeted genes and the greater degeneracy of the corresponding miRNA. Strikingly, A, B, C ratios are much higher 7.2, 134.7, 2.1, respectively in the module 2 (red module in Fig. 2), suggesting greater pluripotentiality of these miRNAs. This result demonstrates that every single miRNA in that module targets a large number of protein-coding genes. Similarly, hub genes–genes known to be targets of a large number of miRNAs [32]—are less numerous in this module. The module 2 acts mainly on genes involved in signal transduction and is able to regulate through pluripotentiality a myriad of different signals from the environment either in the course of development or in response to stresses or environmental changes. In contrast, the module 1 (blue module in Fig. 2), acting mainly on transcriptional regulation, shows smaller A, B, C ratios: 1.1, 58.1, 0.7, respectively, thus suggesting greater degeneracy to fan in signals to key transcriptional factors.

**Table 1:** Characteristics of the different modules in the miRNA network (Fig. 2)

Characteristics	Module 1	Module 2
Main GO term	Transcription regulation	Signal transduction
Number of miRNAs	323	132
Number of links	1,938	407
Average number of targets	3,873	3,508
Number of unique targets	18,762	17,780
Number of non-redundant targets	362	946
Number of generic targets	231	270
Number of hub genes	142	77
Ratio A (# non redundant targets/miRNA)	1.1	7.1
Ratio B (# unique targets/miRNA)	58.1	134.7
Ratio C (# generic targets/miRNA)	0.7	2.1

In-silico analysis of miRNA predicted targets was performed with the DIANA-microT v3 algorithm. The number of unique targets is the number of different genes that are regulated by at least one miRNA in the module. The non-redundant targets are genes that are targeted by only one miRNA of the module (target genes not shared by any other miRNAs). Generic targets are those that are targeted by all miRNAs of the module (e.g, 132 miRNAs in the module 2). Gene hubs are the generic targets of each module that are also target hubs, that is, genes that are highly targeted by the whole miRNome [1, 32]

# stands for “number of”

## The miRNA network nests within a Lavallière-tie structure

The bow-tie structure of networks first proposed by Csete and Doyle in 2004 (Fig. 1) was an important step toward higher-resolution modeling of complex biologic processes. Here, we propose to integrate signal transduction and non-coding RNA (ncRNA) to complete the organizing principle of gene expression network. Indeed, signaling networks and epigenetic factors fan in information from the cellular microenvironment through receptors, kinase-signaling cascades or secondary messengers (signal transduction), to act on Trans* (transcription/translation) machinery through regulation of transcriptional activators or repressors and thereby cope with developmental programs or microenvironmental changes that direct cells to a new fate. Non-coding RNA (ncRNA), including miRNAs, are genes that are transcribed but not translated, which play a major role in regulation of gene expression [12].

**Fig. 3:**
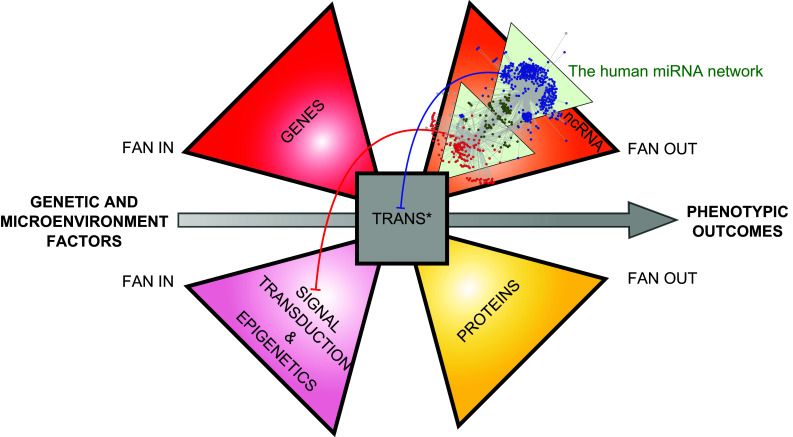
Lavallière-tie structure to model the integration of the miRNA network into a larger gene expression network. The miRNA network is nested within a noncoding RNA (ncRNA) wing and regulate gene expression by acting both at the signal transduction level (*red arrows*) and at the Trans* (transcription/translation) machinery level (*blue arrow*) through feedback loops. The core module of the Lavalliere-tie is composed of few polymerases and universal components (nucleotides, aminoacids, ribosomes units) allowing transcription of both coding and non-coding genes and translation of protein coding genes. While three wings of the structure are composed of molecular objects, the fourth wing manages the flow of information coming from the microenvironment of the cell, through signal transduction and epigenetics mechanisms

We propose a new architecture integrating the flow of information via signal transduction, as well as the flow of material and energy. This organization enables the quick adjustment of either ncRNAs or protein supply and demand to spatio-temporal fluctuation in relation with the genetic program and the microenvironment of the cell. This network possesses the specific structure of a “Lavallière-tie”—a distinct tie knot that is similar to a bowtie but with three or four wings (Fig. 3). Similar to the bowtie structure, the Lavallière-tie architecture contains a small conserved core of common elements. We believe that the miRNA network that we have characterized is integrated into this gene expression network (Fig. 3).

As already mentioned, the core of the tie is a small knot made of a few universal polymerases and a minimal set of building blocks (ribonucleotides and amino acids) acting as common currencies, which confer robustness to the Lavallière-tie structure. Indeed, these shared currencies favor the exquisite adjustment of supply and demand with the minimal enzyme synthesis. However, this core that creates robustness can also reveal fragility. Thus, it is not surprising that RNA polymerase dysfunction ranks among the most important causes of carcinogenesis [2] or that viral pathogens hijack signaling pathways and transcription factors for their own survival [30].

The functions of miRNAs are broadly classified into two categories: setting the mean of the expression level of the target genes (referred to as expression “tuning”) and reducing their expression variance (expression “buffering” or “homeostasis”). Although independent, these two functions are not mutually exclusive. In the transcription network, transcription factors (TFs) and miRNAs may act complementarily. In terms of expression, TFs may play a dominant role in setting the mean level of expression. The task of keeping the system close to that mean during development or in response to stress requires the participation of miRNAs.

By acting as the brakes on Trans* machineries, the miRNA network plays a crucial role in regulating the core of the structure (Fig. 3). Martinez et al. have suggested that miRNAs complete TFs in forming feedback loops [27] on gene expression. Regarding the miRNA network, we believe that the degeneracy of the module 1 (acting primarily on transcriptional regulation) and the pluripotentiality of the module 2 (acting primarily on signal transduction) work in concert to maintain the mean expression level of a given gene as well as to exquisitely buffer its expression (Fig. 3). Whatever the spatio-temporal variation in the expression of these miRNAs, over the course of development or according to a specific location within an organ, in response to a stress or a new challenge, degeneracy and pluripotentiality would enable miRNAs to adjust the expression level of target genes and to thereby act as shock absorbers. This hypothesis might be verified by considering whether, under different conditions (different developmental stages, time-course response to stress, mutated vs non mutated cells, healthy vs pathological cells), the miRNA expression fluctuates more than that of their target genes. More precisely, we hypothesize that degeneracy participates in tuning gene expression, while pluripotentiality buffers the expression of targets, thus providing more robustness to the system. Again, this hypothesis could be verified by analyzing the degeneracy and pluripotentiality properties of miRNAs that are involved mainly in developmental processes in contrast to those involved in stress responses in differentiated or adult tissues. It is interesting to note that genes with specific tissue expression patterns tend to present a longer 3′UTR with more miRNA binding sites [33] and that the expression of miRNAs increases and diversifies during embryonic development [34].

Nearly 60 years ago, Waddington formulated the concept of canalization of a developmental program. Indeed, similar to water in a valley that always flows in a stable path, the acquisition of a phenotype over the course of development is a robust and canalized process [38]. As we previously mentioned in the introduction, extensive studies have shown that miRNAs in humans have extreme functional importance as well as functional dispensability [37]. Facing these paradoxical properties, Wu et al. proposed that miRNAs might play another role beyond the conventional regulatory function, such as phenotypic canalization [4, 5, 27, 36, 42]. In the same vein, we suggest that miRNA degeneracy and pluripotentiality properties, within a lavallière-tie organizational structure, would confer robustness to gene expression network and would participate in phenotypic canalization.
